# Physical activity and quality of life of children and adolescents with juvenile idiopathic arthritis, juvenile systemic lupus erythematosus and juvenile dermatomyositis during the COVID-19 pandemic

**DOI:** 10.1016/j.jped.2025.01.011

**Published:** 2025-04-01

**Authors:** Renata Soares, Fabiana de Carvalho Silva, Jade Dib Fernandez, Melissa Mariti Fraga, Maria Teresa Terreri, Claudio Arnaldo Len

**Affiliations:** Universidade Federal de São Paulo (UNIFESP), Escola Paulista de Medicina (EPM), Departamento de Pediatria, Divisão de Reumatologia Pediátrica, São Paulo, SP, Brazil

**Keywords:** Sedentary lifestyle, Pandemic, Quality of life, Fatigue, Infancy, Rheumatic diseases

## Abstract

**Objectives:**

1) To assess the level of physical activity of children and adolescents with IMRD (juvenile idiopathic arthritis – JIA, juvenile systemic lupus erythematosus—JSLE, or juvenile dermatomyositis - JDM) throughout the COVID-19 pandemic in a tertiary reference service, and 2) To assess the HRQoL and fatigue in these patients.

**Methods:**

The authors included 57 children and adolescents with JIA, JSLE, and JDM, who were clinically inactive according to the assisting physician evaluation. The control group consisted of healthy children. Data was collected during the period of social isolation. The instruments used for the assessments were the International Physical Activity Questionnaire (IPAQ), the Pediatric Quality of Life Inventory 4.0 (PedsQL 4.0), and the Pediatric Quality of Life Inventory – Fatigue Module (PedsQL – Fatigue Module).

**Results:**

About 68.5 % of patients and 79.3 % of controls were considered active regarding physical activity, without any difference between physical activity intensity scores between the groups. Regarding HRQoL, the authors observed lower scores in patients' physical, social, and school functioning domains. The authors observed that patients had higher levels of fatigue according to parents and caregivers.

**Conclusion:**

The impact on physical activity levels of children and adolescents with IMRD throughout the COVID-19 pandemic was positive, with the majority of patients being classified as active, according to the IPAQ questionnaire. Furthermore, the patients engaged in moderate and light physical activities, similar to healthy controls. Regarding HRQoL, the present data showed that patients had lower scores in most of the dimensions assessed.

## Introduction

Immune-Mediated Rheumatic Diseases (IMRD) are chronic conditions and are related to alterations in both innate and adaptive immunity. The most frequent IMRD in childhood and adolescence are juvenile idiopathic arthritis (JIA), juvenile systemic lupus erythematosus (JSLE), and juvenile dermatomyositis (JDM). These diseases affect the musculoskeletal system in various ways and with varying intensity. Those children and adolescents usually have a decreased volume of physical activity.^1^^,^^2^ The treatment for these manifestations consists of the appropriate use of medication and physical rehabilitation, including exercises, physiotherapy, and other modalities.^1^^,^^3^, ^4^, ^5^

Recent studies on the impact of the COVID-19 pandemic have shown an even greater reduction in levels of physical activity in children and adolescents with IMRD,^6^, ^7^, ^8^ with a negative impact on physical and mental health.^4^^,^^6^^,^^8^ On the other hand, no greater risks of infection, or complications from SARS-CoV-2 infection, have been observed. Social isolation during the pandemic was undertaken, as recommended by pediatricians and pediatric rheumatologists.^9^ This intensified some aspects related to Health-Related Quality of Life (HRQoL). For example, decreases in physical activity levels are not solely due to clinical manifestations of the diseases, but also result from social isolation.^2^^,^^10^

Sedentarism, which had already been observed in the pediatric age group, resulting from various factors, became even more evident during the COVID-19 pandemic.^6^^,^^7^^,^^11^ Intending to minimize sedentarism during this period, the World Health Organization (WHO) recommended that, as far as possible, a series of measures should be adopted, such as doing physical exercises at home, outdoor walks, and some lifestyle changes, with emphasis on movement.^11^

In the present study, conducted throughout the COVID-19 pandemic, the authors sought to measure the levels of physical activity, HRQoL^12^ and fatigue^13^ in patients with JIA, JSLE, and JDM, to evaluate the real impact of social isolation on these patients when compared to healthy children and adolescents.

## Methods

In this descriptive cross-sectional study, patients of both sexes, aged between 7 and 17 years, with a diagnosis of JIA, JSLE, or JDM, diagnosed according to current criteria,^14^, ^15^, ^16^ and regularly followed up in a Pediatric Rheumatology service, were included.

Both patient selection and assessment in the study were consecutive and according to the appointment schedule. Inclusion criteria were not presented on the day of evaluation signs, symptoms, or laboratory tests indicative of clinical or laboratory activity of their diseases for at least 6 months, as per medical evaluation, and not having presented general health complaints that had prevented any type of physical activity in the last 30 days, such as infectious diseases or traumas according to the information.

The control group consisted of healthy children and adolescents, of both genders, aged between 7 and 17 years, enrolled in two schools in the municipalities of Barueri/SP and Santana de Parnaíba/SP. Data was collected between May 2021 and October 2021, during the COVID-19 pandemic period. During that time, social isolation was total, following public health recommendations. Both patients and controls were not attending schools, clubs, or other socio-educational activities.

Patients and controls were assessed on a single occasion, through in-person or telephone interviews, conducted by the principal researcher. The assessment of disease activity was based on medical record data and in the opinion of the attending physician.

Patients' demographic data (age and sex) and baseline diagnosis were collected from medical records. As for controls, demographic data including age and sex was obtained through interviews with caregivers/parents. The participation of patients and healthy controls was voluntary. All parents and patients completed informed consent and assent forms. The present study was approved by the local Ethics and Research Committee (ERC).

### Questionnaires

To assess the level of physical activity the authors used The International Physical Activity Questionnaire (IPAQ),^17^ proposed by the World Health Organization in 1998 to serve as a global assessment tool and determine the level of physical activity.^17^ It has two forms: short and long. In this study, the authors chose to use only the short form, as most outdoor activities were suspended due to the period of social isolation during the COVID-19 pandemic. This questionnaire classifies each subject in the study population as: very active, active, irregularly active, or sedentary.^17^

A "very active" individual is someone who met the recommendations for vigorous activities (5 or more days per week with 30 min or more per session, or 3 or more days per week for 20 min or more per session combined with moderate physical activity and/or walking for 5 or more days per week for 30 min or more per session).^17^ An "active" individual needs to meet the recommendations of 3 or more days per week and 20 min or more per session of vigorous physical activity, moderate physical activity, or walking for 5 or more days per week with 30 min or more per session, or 5 or more days per week and 150 min or more per week of walking combined with moderate physical activity, combined with vigorous physical activity.^17^ An "irregularly active" individual engages in physical activity but is insufficient in terms of frequency or duration. This group can be divided into two subgroups: insufficiently active A (meets one of the criteria; either in terms of frequency or in terms of duration) and insufficiently active B (does not meet either the frequency or duration criteria).^17^ A "sedentary" individual is someone who does not engage in any physical activity for at least 10 continuous minutes during the week.^17^ This questionnaire was applied to each patient and each control.

The generic questionnaire PedsQL 4.0 was developed to measure HRQoL.^12^ It comprises 4 domains: physical with 8 items, emotional with 5 items, social with 5 items, and school functioning with 5 items; it consists of self-assessment by the child or adolescent and the caregiver's report. The items are identical for children or adolescents and for the primary caregiver. Responses are classified as: never, almost never, sometimes, often, and almost always.^12^

The PedsQL Fatigue Module is an instrument that measures fatigue and tiredness.^13^ It has 3 domains: general fatigue or tiredness, with 6 items, fatigue or tiredness related to sleep and rest, with 6 items, and cognitive fatigue or mental tiredness, with 6 items. This questionnaire comprises self-assessment by adolescents aged 13 to 18 and the caregiver's report for all ages. The items are identical for children or adolescents and for the primary caregiver.^13^

### Sample size calculation

A sample plan was conducted based on data from Mendonça et al.^1^ and Matsudo et al.^17^ establishing a sample size of 114 individuals, with 57 in the patient group (distributed as follows: 19 patients with JIA, 19 patients with JSLE, and 19 patients with JDM) and 57 in the control group. For calculation, a sampling error of 0.2 % and an alpha error of 5 % were adopted, using power analysis and a sample size system from the SPSS 20.0 statistical package.

### Statistical analysis

The calculations presented in this report were performed using software R 4.2.0 (R Core Team, 2022), and the graphs were constructed using the ggplot2 package (Wickham, 2009). Descriptive statistics for quantitative variables were compiled using means and standard deviations, represented by mean ± SD or medians, and interquartile ranges represented by median when the data distribution did not follow the normal distribution, assessed by using the Kolmogorov-Smirnov test.

For qualitative variables, absolute and relative frequencies were considered, presented as n (%). A significance level of 5 % was adopted for statistical tests, and all tests were considered bilateral.

For the comparison of quantitative variables, *t*-tests or Wilcoxon-Mann-Whitney tests were used. Comparisons between the two groups for categorical variables were performed using Fisher's exact test. To verify the relationship between two numerical variables, Spearman correlation coefficients (ρ) were calculated.

## Results

A total of 57 patients and 58 controls were included. The patient group had a mean age of 13.4 ± 2.8 years, while the control group had a mean age of 12.0 ± 2.6 years (*p* = 0.008). Regarding the sex of the patients, 32 (63.1 %) were female; for the controls, 33 (56.9 %) were female. Concerning the diagnoses of the 57 patients included in the study, 19 had JIA, 19 had JSLE, and 19 had JDM, all of them with their diseases clinically inactive for at least 6 months and receiving prescribed treatments according to current recommendations for each disease, according to the evaluation of the attending physician.

One shows the classification of patients and controls regarding the frequency of physical activity according to the IPAQ. The authors did not observe a statistically significant difference between the groups when considering IPAQ scores overall. Most patients and controls were considered active, with no difference between the groups (*p* = 0.338).

Table 1 also displays IPAQ scores concerning the intensity of physical activity. Overall, the authors did not observe any differences in intensity between the two groups (*p* = 0.155). However, when classifying the intensity of physical activities as vigorous, moderate, and light, the authors observed that no patients engaged in vigorous physical activities, while 41/58 controls (70.6 %) performed such activities at some point during the pandemic (*p* = 0.042). Regarding moderate and light activities, the authors did not observe any differences between the groups (*p* = 0.122 and *p* = 0.748, respectively). Only one patient was classified as sedentary, and no controls received this classification.

**Table 1:** International Physical Activity Questionnaire (IPAQ) applied in patient and control groups (frequency and intensity).

	Patients (*n* = 57)	Controls (*n* = 58)	Total (*n* = 115)	*p*-value
				
IPAQ classification				
IPAQ–physical activity frequency				
Total–active	56 (98.2 %)	58 (100 %)	114 (99.1 %)	0.338^a^
Very active	12 (21.1 %)	18 (31.0 %)	30 (26.1 %)	–
Active	27 (47.4 %)	28 (48.3 %)	55 (47.8 %)	–
Irregularly active A	11 (19.3 %)	5 (8.6 %)	16 (13.9 %)	–
Irregularly active B	6 (10.5 %)	7 (12.1 %)	13 (11.3 %)	–
Sedentary	1 (1.8)	0 (0.0 %)	1 (0.9 %)	–
IPAQ–physical activity intensities				
IPAQ–score (MET)	1311 [600;3390]	2035 [870;3449]	1680 [720;3441]	0.155^b^
IPAQ vigorous activity (MET-minutes/week)	0.0 [0;1200]	680 [0;1860]	400 [0;1520]	0.042^b^
IPAQ moderate activity (MET-minutes/week)	720 [280;1200]	840 [420;1500]	720 [340;1440]	0.122^b^
IPAQ light activity (MET-minutes/week)	165 [0;330]	116 [0;335]	132 [0;330]	0.748^b^

IPAQ, International Physical Activity Questionnaire; MET, Metabolic Equivalent of Task.

a Measurements presented as n (%) (absolute frequency and percentage relative to the group) and *p*-value relative to the Chi-square test.

b Measurements presented as median [Q1; Q3] (median and interquartile range) and *p*-value relative to the Wilcoxon/ Mann-Whitney test.

In Table 2, the authors present the results of the PedsQL 4.0 questionnaire when applied to patients and controls in the physical, emotional, social, and school functioning dimensions. Overall, questionnaire scores were higher for controls in all dimensions. Statistically significant differences were observed in the total PedsQL 4.0 scores between the two groups, as well as in the physical and school functioning dimensions, both from the perspective of parents/caregivers (*p* < 0.001) and children/adolescents (*p* = 0.011), in both dimensions. On the other hand, in the emotional dimension, the authors did not observe any statistically significant differences between the two groups from the perspectives of parents/caregivers or children/adolescents. In the social dimension, the authors observed a statistically significant difference between the two groups only from the perspective of parents/caregivers (*p* < 0.001).

**Table 2:** Pediatric Quality of Life Inventory 4.0 (PedsQL 4.0) questionnaire for patients and controls.

	Patients (*n* = 57)	Controls (*n* = 58)	Total (*n* = 115)	*p*
PedsQL 4.0				
				
PedsQL 4.0–parents	68.5 [50;81.8]	83.8 [73.1;90.2]	77.7 [63.1;85.9]	< 0.001^b^
PedsQL 4.0–parents (physical)	70.3 [45.3;87.5]	93.8 [81.2;100.0]	82.8 [62.5;96.9]	< 0.001^a^
PedsQL 4.0–parents (emotional)	60,0 [43.8;76.2]	70.0 [55.0;80.0]	65.0 [50.0;80.0]	0.066^b^
PedsQL 4.0–parents (social)	75.0 [63.8;90.0]	92.5 [80.0;100.0]	85.0 [70.0;100.0]	< 0.001^a^
PedsQL 4.0–parents (school functioning)	62.5 [45.0; 75.0]	85.0 [65.0;95.0]	70.0 [55.0;85.0]	< 0.01^b^
PedsQL 4.0–children	72.8 [57.6;80.4]	77.2 [68.5;84.8]	75.0 [63.0;82.1]	0.011^b^
PedsQL 4.0–children (physical)	75.0 [62.5;87.5]	84.4 [78.1;93.0]	81.2 [67.7;90.6]	0.002^a^
PedsQL 4.0–children (emotional)	65.0 [45.0;80.0]	60.0 [45.0;80.0]	60.0 [45.0;80.0]	0.848^b^
PedsQL 4.0–children (social)	80.0 [60.0;95.0]	85.0 [70.0;95.0]	80.0 [65.0;95.0]	0.056^b^
PedsQL 4.0–children (school functioning)	65.0 [50.0;80.0]	80.0 [65.0;90.0]	75.0 [60.0;85.0]	< 0.001^b^

PedsQL 4.0, Pediatric Quality of Life Inventory 4.0.

a Measurements are presented as median [Q1; Q3] (median and interquartile range) and *p*-value relative to the Wilcoxon-Mann-Whitney test.

b Measurements presented as median [Q1; Q3] (median and interquartile range), but where distribution is normal, so the test conducted for comparison was the *t*-test.

Regarding the PedsQL - Fatigue Module questionnaire, the authors observed that patients exhibited higher levels of fatigue from the perspective of parents/ caregivers. Table 3 shows that scores were higher for the control group, indicating lower fatigue, in the total score (*p* = 0.007), in the fatigue-tiredness score (*p* = 0.005), and in the fatigue-sleep score (*p* = 0.001).

**Table 3:** Pediatric Quality of Life Inventory - Fatigue Module questionnaire for patients and controls, from the perspective of parents/caregivers.

PedsQL – Fatigue Module	Patients (*n* = 57)	Controls (*n* = 58)	Total (*n* = 115)	*p*-value
Total	69.0 ± 18.2	78.3 ± 17.2	73.8 ± 18.2	**0.007**^a^
Tiredness	69.5 ± 20.4	80.2 ± 19.2	75.0 ± 20.4	**0.005**^a^
Sleep	65.4 ± 24.9	79.8 ± 19.7	72.8 ± 23.4	**0.001**^a^
Mental	75.8 ± 19.8	77.2 ± 22.4	76.5 ± 21.1	0.489^b^
PesdQL: *Pediatric Quality of Life Inventory*				

a Measurements are presented as mean ± standard deviation and *p*-value relative to the *t*-test.

b Measurements presented as mean ± standard deviation, but where distribution is not normal, the test conducted for comparison was the Wilcoxon-Mann-Whitney.

## Discussion

In this study, the authors assessed three parameters related to the daily life of a group of patients with IMRD aged 7 to 17 years during the COVID-19 pandemic, followed at a pediatric rheumatology tertiary outpatient clinic: levels of physical activity, health-related quality of life (HRQoL), and fatigue. For this purpose, the authors used validated instruments for this setting, which were also applied to healthy children and adolescents.^18^ Regarding levels of physical activity, according to the IPAQ^17^ questionnaire, the authors did not observe significant differences between patients and controls. Regarding HRQoL, measured by the generic PedsQL 4.0^12^ questionnaire, and fatigue, measured by the PedsQL - Fatigue Module^13^ instrument, the authors found generally lower scores for the patient group. However, the present findings are similar to other measurement studies conducted before the pandemic.^1^^,^^12^^,^^13^

The COVID-19 pandemic had a major impact on the routines of children and adolescents in almost all countries, especially due to the need for social isolation in schools and even in open areas such as public squares and parks.^11^^,^^18^ Milatz et al.^19^ followed a group of patients with JIA and a control group for 2 years during the COVID-19 pandemic. They observed that initially, the physical activity levels between the two groups were significantly different, with patients being less physically active than their healthy counterparts. However, over the course of the follow-up, the proportion of patients with JIA who did not engage in regular physical activity decreased, while the proportion of healthy peers without regular physical activity increased.

Another interesting finding was how scores from tools like the PedsQL converged. As a result, there were no significant differences in general levels of physical activity between the groups. This suggests that children and adolescents with JIA initially have a lower level of physical activity, but with time, through guidance and simple measures—such as responding to questionnaires that prompt reflection on their sedentary behavior—they may be influenced to engage more in physical activity. In another study, Milatz et al.^20^ observed that children and adolescents with JIA were equally likely to achieve the physical activity levels recommended by the WHO, underscoring the importance of encouraging them to adopt this practice.

Children and adolescents with IMRD are naturally less active than healthy children.^9^ Moreover, the clinical manifestations of these diseases are associated with a more sedentary lifestyle, which can exacerbate symptoms and trigger a cycle of physical inactivity.^4^^,^^21^, ^22^, ^23^

In the case series, the majority of patients and controls engaged in moderate and light physical activities, with no statistically significant difference between the two groups. In other studies, reported in the literature during the pandemic, there is no reference to the intensity of physical activities. Undoubtedly, there was concern among professionals about the potential decrease in physical activity levels of patients with IMRD during the pandemic. In the outpatient clinic, the authors tried to encourage patients to move as much as possible during the pandemic, whether through guidance in face-to-face consultations, telemedicine, or face-to-face or online appointments with the team of physiotherapists. Telerehabilitation was conducted in individual or group sessions, according to the need and internet access of the families. Mendonça et al.^1^ applied the Pilates method to a group of patients with JIA and observed an improvement in their HRQoL during the course of treatment. In a recent review article, Rochette et al.^4^ emphasize the importance of regular physical activity for patients with JIA, aiming at symptom relief, and improvement of sleep, mental health, and quality of life.

Children and adolescents with IMRD generally have lower HRQoL scores than healthy individuals of the same age.^12^^,^^23^^,^^24^ It is necessary to measure some dimensions related to HRQoL, such as levels of physical activity and fatigue, among others. Several instruments have been developed for measuring these aspects of patients' and healthy individuals' daily lives.^12^, ^13^, ^14^ These tools were designed for longitudinal measurement, especially when individuals are exposed to lifestyle changes or treatments to improve their diseases. Examples of these instruments used in the routine follow-up of patients with IMRD, translated and validated for the setting, are the International Physical Activity Questionnaire (IPAQ),^17^ the Pediatric Quality of Life Inventory 4.0 (PedsQL 4.0),^12^ and the Pediatric Quality of Life Inventory - Fatigue Module (PedsQL - Fatigue Module).^13^ All these instruments have been applied in patients with IMRD and, generally, have shown lower scores for patients compared to healthy individuals.

In this study, the authors observed that this reality was also evident during the pandemic. PedsQL 4.0 scores were lower for patients than for controls in the physical, social, and school functioning dimensions from the perspective of parents/caregivers, and in the physical and school functioning dimensions from the perspective of children and adolescents.

The present study had some limitations. The authors were unable to match patients and controls by age. Despite the efforts to include controls matched by age, the authors encountered significant difficulty in recruiting healthy controls, as data collection was conducted during the peak of social isolation during the pandemic. The authors also had to have convenience sampling in IMRD patients, and it might reduce the generalizability of the findings in this study.

However, the authors believe that this fact does not interfere with the results, as the physical activity level of children and adolescents with mean ages of 13.4 years (± 2.8 years) for patients and 12.0 years (± 2.6 years) for controls is similar in daily life. Another limitation is the collection of physical activity data through a questionnaire (IPAQ). The authors attempted to use an accelerometer in the initial stages of the study, but the reduction in the number of visits to the outpatient clinic for device collection precluded the use of this method, which is the gold standard for measuring levels of physical activity.

## Conclusion

In the present study, the authors observed that the impact on levels of physical activity in children and adolescents with IMRD during the COVID-19 pandemic was positive, with the majority of patients being classified as active according to the IPAQ questionnaire. Furthermore, the patients engaged in moderate and light physical activities, similar to healthy controls.

Regarding HRQoL, these results showed that the scores on the generic PedsQL 4.0 questionnaire during the pandemic were lower than those observed in healthy controls in the physical, social, and school functioning dimensions. Fatigue scores in total, tiredness, and sleep dimensions were also lower in patients with IMRD compared to controls.

These findings, which have been described during the pandemic, compel us to take action to develop a more holistic care model, in which patients should not only be classified as active or inactive from a clinical or laboratory standpoint but also assessed and assisted from emotional and social perspectives.

## Financial support

CNPq/CAPES Master's scholarship – number 23.990.

## Authors’ contributions

Renata Soares: Involved in the conception and design of the study; acquisition of data; analysis and interpretation of data; drafting the article and revising it critically for important intellectual content; and in the final approval of the version to be submitted.

Fabiana de Carvalho Silva: Involved in the conception and design of the study; acquisition of data; and in the final approval of the version to be submitted.

Jade Dib Fernandez: Analysis and interpretation of data; drafting the article and revising it critically for important intellectual content; and in the final approval of the version to be submitted.

Melissa Mariti Fraga: Drafting the article and revising it critically for important intellectual content; and in the final approval of the version to be submitted.

Maria Teresa Terreri: Analysis and interpretation of data; drafting the article and revising it critically for important intellectual content; and in the final approval of the version to be submitted.

Claudio Arnaldo Len: Involved in the conception and design of the study; acquisition of data; analysis and interpretation of data; drafting the article and revising it critically for important intellectual content; and in the final approval of the version to be submitted.

## Conflicts of interest

The authors declare no conflicts of interest.
